# How carvedilol does not activate β_2_-adrenoceptors

**DOI:** 10.1038/s41467-023-42848-5

**Published:** 2023-11-30

**Authors:** Robert J. Lefkowitz, Howard A. Rockman, Paul J. Shim, Samuel Liu, Seungkirl Ahn, Biswaranjan Pani, Sudarshan Rajagopal, Sudha K. Shenoy, Michel Bouvier, Jeffrey L. Benovic, Stephen B. Liggett, Robert R. Ruffolo, Michael R. Bristow, Milton Packer

**Affiliations:** 1 Department of Medicine, Duke University, Durham, NC USA; 2 Howard Hughes Medical Institute, Duke University, Durham, NC USA; 3 Department of Biochemistry, Duke University, Durham, NC USA; 4 Department of Biochemistry and Molecular Medicine, Université de Montréal, Montreal, Quebec Canada; 5 Department of Biochemistry and Molecular Biology, Thomas Jefferson University, Philadelphia, PA USA; 6 Departments of Molecular Pharmacology and Medicine, University of South Florida College of Medicine, Tampa, FL USA; 7 Research & Development, Wyeth Pharmaceuticals, Philadelphia, PA USA; 8 Department of Medicine, Division of Cardiology, University of Colorado - Anschutz Medical Campus, Aurora, CO USA; 9 Baylor University Medical Center, Dallas, TX USA; 10 Imperial College, London, UK

**Keywords:** Drug discovery, Cell biology, Cardiovascular biology

**arising from** T. Benkel et al. *Nature Communications* 10.1038/s41467-022-34765-w (2022)

The introduction of β-adrenergic receptor blockers, “β-blockers”, as a treatment for heart failure decades ago was a major advance in cardiovascular medicine^1–4^. Moreover, it has been clear for a number of years that some β-blockers with “intrinsic sympathomimetic activity” (ISA) due to weak partial agonist activity mediated through G protein stimulation did not share this salutary effect^5,6^. Yet, a recent paper by Benkel et al. ^7^, “How carvedilol activates β_2_-adrenoceptors”, using engineered cells that lack either G proteins or arrestins, proposes that carvedilol, a widely used and effective β-blocker for heart failure, signals through β_2_AR-mediated G protein activation. Here, we explain why we believe the conclusions put forward by Benkel et al. are incorrect and certainly cannot be extended to clinical implications and should not guide decisions in the clinic.

Contrary to the claims of Benkel et al.^7^, it has previously been established that in systolic heart failure (currently known as Heart Failure with reduced Ejection Fraction, HFrEF), β-blockers without ISA have beneficial effects whereas those with ISA do not^5,6^. None of the three FDA-approved β-blockers for the treatment of heart failure (carvedilol, metoprolol and bisoprolol) and nebivolol (approved in Europe) are classified as having ISA^8^. This is in concert with clinical trial evidence that drugs such as milrinone and ibopamine, which enhance cyclic AMP (as is the case for β-blockers with ISA), have deleterious effects in systolic heart failure^9,10^. Benkel et al. cite one post-myocardial infarction trial^11^ of a β-blocker with ISA. Yet when considering the 25 post myocardial infarction β-blocker trials carried out in the 1970’s and 1980’s, those with ISA showed substantially less beneficial, or even negative effects on patient outcomes^12^.

It has been established that cells such as those used in most of the studies in Benkel et al., which have been transfected with robust expression vectors consisting of cDNAs encoding GPCRs (in this case the β_2_ adrenoceptor, β_2_AR), express receptors at levels that are generally at least 10- to 100-fold higher than the levels of endogenous expression^13^. Benkel et al. do not directly report the level of receptor expression in their cells. It can be readily demonstrated that in untransfected HEK-293 cells, which express low (physiologic) levels of endogenous β_2_ARs, carvedilol does not stimulate an elevation in the levels of cyclic AMP^14^, and this is true even in some systems with high level overexpression of β_2_ARs^15^. Not until receptor levels are dramatically raised by overexpression, and at high concentrations of carvedilol, is very low-level stimulation observed. Indeed, the authors themselves point out “…that the majority of assays to disentangle G-protein versus Arrestin biased signaling were performed in overexpression systems. Hence extrapolation of our data to primary cells or the in vivo situation must be performed with caution…”. The concentrations of carvedilol used in Benkel’s overexpressing cells, 10 micromolar for many studies and above one micromolar in most, are well above the plasma levels the drug ever achieves clinically. For example, maximal plasma levels of carvedilol after a typical 50 mg dose in humans are well below 100 nM, of which 95 to 98% is protein bound, meaning the effective therapeutic concentration is, in fact, an order of magnitude lower^16,17^.

Our review of the literature finds at least 9 papers in which more physiological cardiovascular systems were used to test whether carvedilol possesses ISA^18–26^. These studies used a wide array of in vivo and ex vivo cardiac preparations ranging from in vivo pithed rats^24,25^ to isolated myocytes^21,24,26^ to isolated atrial and ventricular myocardial preparations^18–20,22,23^, from multiple species including guinea pig^26^, mouse^21^, rat^18,24,25^, and human failing and non-failing myocardium^18–20,22,23^. In no case was any stimulatory effect seen with carvedilol at the level of cyclic AMP^18,19,24,26^, isolated muscle force generation^19,20,22,23^, contractility^21^ or beating rate^24,25^. None of these studies were cited in Benkel et al.^7^, and all failed to show that carvedilol demonstrates ISA in cardiac preparations with endogenous levels of adrenergic receptor expression. These results are in conflict with the findings reported by Benkel et al. in the set of experiments in which isolated neonatal mouse cardiac myocytes are utilized (Fig. 3 in Benkel et al.). Additionally, in our view, several of the papers that are referenced in support of carvedilol possessing ISA are cited misleadingly. For example, Benkel et al. cite reference 24 as stating that carvedilol binding is sensitive to GTP^27^. While it is true that the Maack et al. paper claimed that carvedilol has ISA, in fact, their data showed that carvedilol has no ISA in 6 out of 7 experiments performed^27^. Other such papers cited either explicitly state that “carvedilol has no intrinsic sympathomimetic activity“^28^, make no statements about ISA^29^, or use non physiologic overexpression systems^30–32^.

Furthermore, the finding that the full agonist isoproterenol and the β-blocker carvedilol through the β_2_AR have quite similar (70%) effect on enhancing heart cell beating rate is inconsistent with fundamental pharmacological principles for the action of a full agonist and a competitive antagonist on receptor activation (Benkel et al., Fig. 3f: + CGP-20712A, 3rd column ~22 bpm, vs. Fig. 3h: + CGP-20712A, 2nd column ~15 bpm). Moreover, the data in Fig. 3 with myocardial cells that indicate the chronotropic effects of isoproterenol and carvedilol are being driven primarily by the β_2_AR is inconsistent with previous studies with β_1_ and β_2_AR knockout mice, both in neonatal cardiac myocytes^33^ and in the intact heart^34,35^ (none cited). These previous studies clearly indicate that such responses to β-agonists are driven either primarily or exclusively by the β_1_AR. Patients who begin taking carvedilol do not develop a tachycardia, and indeed, lowering heart rate in heart failure can be beneficial^36^.

Several laboratories have documented that carvedilol is a weak β-arrestin biased ligand that can stimulate ERK activity in a β-arrestin and/or GRK dependent fashion although most demonstrations have been in the same type of overexpression, high drug concentration systems used here^14,21,37–40^. This understanding of the actions of carvedilol still casts it as a very useful tool for biophysical studies probing the molecular mechanisms of biased signaling. This is evidenced by several studies which demonstrate that the drug stabilizes unique partially active conformations of the β_2_AR^41,42^ with characteristics in common with β-arrestin biased conformations of other GPCRs such as the angiotensin receptor featuring repositioning of transmembrane helix 7 and the short non membrane embedded helix 8^43^. Nonetheless, in Fig. 1 Benkel et al. report that they failed to observe any alteration in carvedilol-stimulated ERK activation in comparing the response between one particular β-arrestin 1/2 double CRISPR knockout HEK-293 cell line and its cognate parental HEK-293 cell line. The authors have published this particular finding before^44^. However, it has been demonstrated by others that independent derivation of such CRISPR β-arrestin knockout lines in two other laboratories leads to cells with very different properties than the ones utilized here^37^ (not cited in this context). For example, in the two distinct parental HEK-293 cells used for derivation of the other two CRISPR knockout lines, transfection with β-arrestin1/2 siRNA led to near elimination of carvedilol-stimulated ERK activation. Moreover, in CRISPR β-arrestin knockout lines derived from these parental cells, no carvedilol stimulation of ERK activity could be demonstrated but could be restored by reintroduction of β-arrestin 1/2 by transfection^37^. These results could not be replicated with the parental and knockout HEK-293 cells used by Benkel et al. These striking differences in β-arrestin signaling amongst the three independently derived CRISPR β-arrestin knockout lines and the parental HEK-293 clones from which they were derived are presumably due to differential “rewiring of the cells” during their derivation and underscore a shortcoming in the Benkel et al. study. Highly engineered systems call for orthogonal approaches to validate the findings, for example using more than one of the published clonal β-arrestin knockout lines and use of siRNA to knock down β-arrestin expression. In fact, in contrast with these variable results obtained with the different CRISPR lines, siRNA mediated reductions in β-arrestin levels in all three parental HEK-293 lines led to consistent reductions in ERK activation not only through the β_2_AR and β_1_AR, but also through the AVP and FSH receptors as well^37^.

In summary, over 30 years of clinical studies support the fact that β-blockers without ISA have beneficial effects, and those with ISA have no benefit or increased mortality in systolic heart failure. The proposal of Benkel et al. that the mechanism responsible for the beneficial actions of carvedilol is ISA is not consistent with the overwhelming evidence that rests securely on decades of basic science studies and numerous clinical trials, and should not guide clinical practice.
